# A New Species of Pengornithidae (Aves: Enantiornithes) from the Lower Cretaceous of China Suggests a Specialized Scansorial Habitat Previously Unknown in Early Birds

**DOI:** 10.1371/journal.pone.0126791

**Published:** 2015-06-03

**Authors:** Han Hu, Jingmai K. O’Connor, Zhonghe Zhou

**Affiliations:** 1 Key Laboratory of Vertebrate Evolution and Human Origin of Chinese Academy of Sciences, Institute of Vertebrate Paleontology and Paleoanthropology, Beijing, China; 2 University of Chinese Academy of Sciences, Beijing, China; Raymond M. Alf Museum of Paleontology, UNITED STATES

## Abstract

We describe a new enantiornithine bird, *Parapengornis eurycaudatus* gen. et sp. nov. from the Lower Cretaceous Jiufotang Formation of Liaoning, China. Although morphologically similar to previously described pengornithids *Pengornis houi*, *Pengornis* IVPP V18632, and *Eopengornis martini*, morphological differences indicate it represents a new taxon of the Pengornithidae. Based on new information from this specimen we reassign IVPP V18632 to *Parapengornis* sp. The well preserved pygostyle of the new specimen elucidates the morphology of this element for the clade, which is unique in pengornithids among Mesozoic birds. Similarities with modern scansores such as woodpeckers may indicate a specialized vertical climbing and clinging behavior that has not previously been inferred for early birds. The new specimen preserves a pair of fully pennaceous rachis-dominated feathers like those in the holotype of *Eopengornis martini*; together with the unique morphology of the pygostyle, this discovery lends evidence to early hypotheses that rachis-dominated feathers may have had a functional significance. This discovery adds to the diversity of ecological niches occupied by enantiornithines and if correct reveals are remarkable amount of locomotive differentiation among Enantiornithes.

## Introduction

Enantiornithes (Aves: Ornithothoraces) represents the dominant Cretaceous clade both in terms of the overall number of specimens and species diversity [1]. More than half this diversity belongs to the Early Cretaceous Jehol Biota in northeastern China [2]. The Huajiying Formation, the first stage of the Jehol Biota [3], is the oldest known enantiornithine bearing deposit in the world [4]. Only three avian fossils have been described, all from the “*Protopteryx*-horizon”: *Eoconfuciusornis* (Confuciusornithiformes), *Protopteryx* (Enantiornithes), and *Eopengornis*, a member of the Pengornithidae [4–6]. The Pengornithidae was considered to be a primitive group and has been resolved as one of the most basal clades of enantiornithines in several phylogenetic analyses [6–10]. This clade is distinct within Enantiornithes, representing the most easily diagnosable clade, characterized by a large number of diagnostic features including: upper and lower jaws with numerous small teeth (over ten in the maxilla alone); scapular acromion process hooked; sternum with single pair of caudal trabeculae (intermediate trabeculae absent); xiphial region of sternum defines wide V (xiphoid process absent); short pygostyle with rounded distal margin; and femur nearly as long as the tibiotarsus [6].

Three specimens are currently referred to this clade: *Pengornis houi* IVPP V15336, *Pengornis* sp. IVPP V18632 (the taxonomic position of which is reassessed here in light of current discoveries), and *Eopengornis martini* STM 24–1. The holotype of *Pengornis houi*, the first described pengornithid, represents the largest known Early Cretaceous enantiornithine [7]. The specimen IVPP V18632 was recently referred to *Pengornis sp*., providing additional anatomical information regarding the sternum and hindlimb of the Pengornithidae, features absent or poorly preserved in *Pengornis houi* IVPP V15336 [10]. These two specimens were collected in the 120 Ma Jiufotang Formation, the last stage of the Jehol Biota, in which avian diversity is highest. The more recently discovered *Eopengornis martini* from the 130.8 Ma Huajiying Formation in Hebei greatly extended the known temporal range of the Pengornithidae, revealing the longest known Early Cretaceous enantiornithine lineage. The holotype specimen of *Eopengornis* preserves a pair of fully pennaceous rachis-dominated feathers, suggesting the ornamental racket-plume morphology in enantiornithines and *Confuciusornis* evolved independently in each lineage [6].

Here we describe a new pengornithid based on a nearly complete specimen preserving integument collected from the Jiufotang Formation near Lingyuan, western Liaoning, for which we erect a new taxon, *Parapengornis eurycaudatus*. This specimen provides new anatomical information that contributes to our understanding of the Pengornithidae, clarifies the taxonomic position of IVPP V18632, previously referred to *Pengornis sp*., and provides evidence that the Pengornithidae may have been a clade of specialized vertical climbers similar to living woodpeckers.

## Materials and Methods

Anatomical nomenclature primarily follows Baumel and Witmer [11].

A new specimen was discovered in the Jiufotang Formation near Lingyuan, western Liaoning of China, then collected and prepared without any artificial modifications by Institute of Vertebrate Paleontology and Paleoanthropology, Chinese Academy of Sciences for its museum collection, and its specimen number is IVPP V18687. We have full permission from the institute, which is represented by Zhonghe Zhou (co-author). No permits are required for the described study, which is based entirely on museum specimens, and the museum collections are accessible to the public; this research complies with all relevant regulations.

Using an expanded and modified version of the O’Connor et al. (2012) [9] dataset we explored the phylogenetic position of *Parapengornis eurycaudatus* through cladistic analysis. The expanded dataset includes *Eopengornis martini* (data from Wang et al. 2014 [6]), the new taxon, and three additional post-cranial characters. A total of 62 taxa were evaluated across 248 characters using TNT [12] (S1 Character List, S1 Dataset).

Institutional Abbreviations: IVPP, Institute of Vertebrate Paleontology and Paleoanthropology, Beijing, China; STM, Shandong Tianyu Museum of Nature, Shandong Province, China.

## Nomenclatural Acts

The electronic edition of this article conforms to the requirements of the amended International Code of Zoological Nomenclature, and hence the new names contained herein are available under that Code from the electronic edition of this article. This published work and the nomenclatural acts it contains have been registered in ZooBank, the online registration system for the ICZN. The ZooBank LSIDs (Life Science Identifiers) can be resolved and the associated information viewed through any standard web browser by appending the LSID to the prefix "http://zoobank.org/". The LSID for this publication is: urn:lsid:zoobank.org:pub: 802441BF-6E87-43C6-99D7-7C3E873503DF. The electronic edition of this work was published in a journal with an ISSN, and has been archived and is available from the following digital repositories: PubMed Central, LOCKSS.

## Results

### Locality and Horizon

Lingyuan, Chaoyang, Liaoning, China, Jiufotang Formation, Early Cretaceous [13].

### Systematic Paleontology

Aves Linnaeus, 1758 [14]

Ornithothoraces Chiappe, 1995 [15]

Enantiornithes Walker, 1981 [16]

Pengornithidae Wang et al., 2014 [6]

*Parapengornis eurycaudatus* gen. et sp. nov.

urn:lsid:zoobank.org:act:023BF172-3901-4C81-8E78-3BBA9DCCED9B

### Holotype

**Fig 1:**
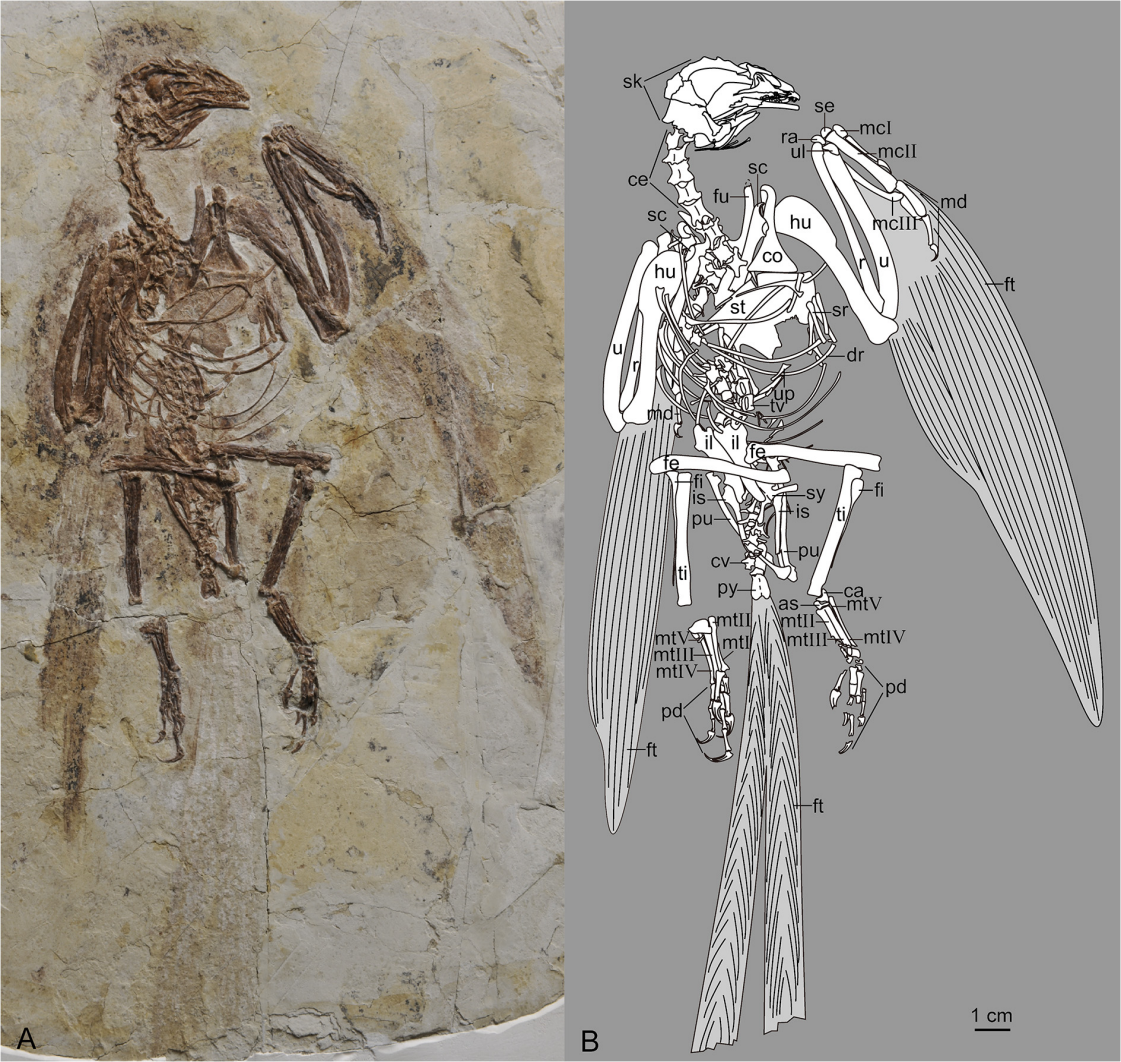
Photograph (A) and camera lucida drawing (B) of IVPP V18687. Abbreviations: as, astragalus; ca, calcaneum; ce, cervical vertebrae; co, coracoid; cv, caudal vertebrae; dr, dorsal rib; fe, femur; fi, fibula; ft, feathers; fu, furcula; hu, humerus; il, ilium; is, ischium; mc I-III, metacarpals I-III; md, manual digits; mt I-V, metatarsals I-V; pd, pedal digits; pu, pubis; py, pygostyle; r, radius; ra, radiale; sc, scapula; se, semilunate carpal; sk, skull; sr, sternal rib; st, sternum; sy, synsacrum; ti, tibia; tv, thoracic vertebrae; u, ulna; ul, ulnare; up, uncinate process.

IVPP V18687 (Fig 1), a nearly complete and articulated individual preserved in a single slab, missing only the distal portion of the sternum and some parts of the left hand and right foot, with impressions of the remiges and rectrices.

### Referred Specimen

IVPP V18632 (a nearly complete articulated skeleton of a subadult individual, formerly incorrectly referred to *Pengornis*).

### Etymology

*Parapengornis* is composed of the Latin prefix ‘para’ and *Pengornis* indicating the close relationship between the new taxon and *Pengornis*. The species name *eurycaudatus*, ‘eury’ means broad and ‘caudatus’ means tail in Latin, indicating the unique broad and laterally expanded pygostyle of the new taxon.

### Diagnosis

A large pengornithid enantiornithine bird characterized by the unique combination of the following features: numerous slender and constricted teeth with pointed and slightly recurved apices; cranial cervicals short but caudal cervicals more elongated; broad pygostyle shorter than half the length of metatarsal III with strongly expanded lateral processes and concave caudal margin (autapomorphy); Y-shaped furcula with slender and straight clavicular rami.

### Description

The specimen is well preserved in nearly full articulation, exposed mostly in dorsal view. It is intermediate in size between *Pengornis houi* and *Pengornis* sp. IVPP V18632 (Table 1; length of femur 39.8 mm in *Parapengornis eurycaudatus*, 48 mm in *P*. *houi*, and 34.8 mm in *Pengornis* sp. IVPP V18632), and thus it is larger than all other previously described Early Cretaceous Enantiornithes, with the exception of *Bohaiornis* LPM B00167, which is approximately equal in size (39 mm) [17].

**Table 1:** Measurements (mm) of IVPP V18687.

Element	Measurement	Element	Measurement
Skull length	39.9	Ischium length (l)	20.7*
Pygostyle length	8.4	Pubis length (l)	37.2
Furcula proximal width	23.4	Femur length (r)	39.8
Hypocleidium length	12.6	Tibia length (r)	40.4
Sternum width	34.2	Fibula length (l)	34.6
Sternum, lateral trabecula length (l)	27.2	Metatarsal I length (l)	8.6
Scapula length (l)	46.3	Metatarsal II length (l)	19.5**
Coracoid length (r)	26.3	Metatarsal III length (l)	20.5**
Humerus length (r)	52.1	Metatarsal IV length (l)	19.1
Humerus, midshaft width (r)	5.2	Metatarsal V length (l)	3.1*
Ulna length (r)	54.9	Pedal phalanx I-1 length (l)	9.2
Ulna, midshaft width (r)	5.2	Pedal phalanx I-2 length (l)	7.4
Radius length (r)	53.7	Pedal phalanx II-1 length (l)	5.2
Radius, midshaft width (r)	2.9	Pedal phalanx II-2 length (l)	7.0
Alular metacarpal length(r)	5.3	Pedal phalanx II-3 length (l)	8.2
Major metacarpal length (r)	24.8	Pedal phalanx III-1 length (l)	6.6
Minor metacarpal length (r)	27.4	Pedal phalanx III-2 length (l)	6.3
Alular phalanx-1 length (r)	11.4	Pedal phalanx III-3 length (l)	6.4
Alular phalanx-2 length (r)	6.7	Pedal phalanx III-4 length (l)	8.2
Major phalanx-1 length (r)	12.7	Pedal phalanx IV-1 length (l)	4.8
Major phalanx-2 length (r)	9.2	Pedal phalanx IV-2 length (l)	2.7
Major phalanx-3 length (r)	4.8	Pedal phalanx IV-3 length (l)	3.2
Minor phalanx-1 length (r)	8.1	Pedal phalanx IV-4 length (l)	4.5
Minor phalanx-2 length (r)	1.2	Pedal phalanx IV-5 length (l)	5.8
Ilium length (r)	24.4*	Tail feather length	131.5*

Abbreviations: l, left; r, right;

* indicates preserved length;

** indicates estimated length.

#### Skull

**Fig 2:**
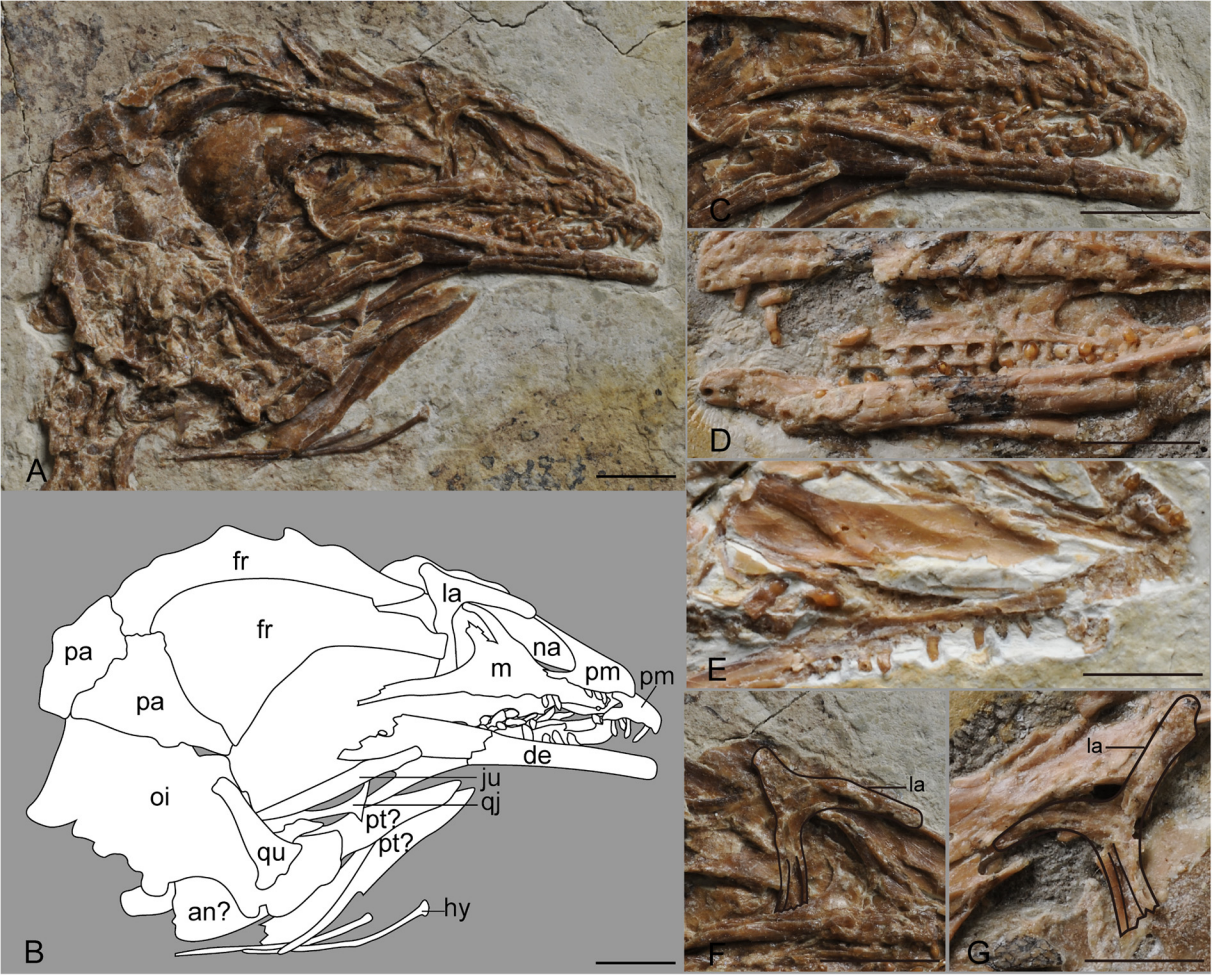
Photograph (A) and camera lucida drawing (B) of the skull of IVPP V18687, and comparison of teeth morphological details of C, IVPP V18687;D, *Pengornis houi*; and E, *Eopengornis martini*, and lacrimals of F, IVPP V18687; and G, *Pengornis houi*. Abbreviations: an, angular; de, dentary; fr, frontal; hy, hyoid; ju, jugal; la, lacrimal; m, maxilla; na, nasal; oi, occipital region; pa, parietal; pm, premaxilla; pt, palatine; qj, quadratojugal; qu, quadrate. Scale bar equals 5 mm.

The skull of *Parapengornis eurycaudatus* (Fig 2A and 2B), preserved in right lateral view, is crushed and some bones are disarticulated from their natural positions, making it difficult to define the shape of the external nares, antorbital fenestra and orbit.

The premaxillae are disarticulated from each other, indicating they were entirely unfused as in *Pengornis houi* [7]. The premaxillary corpus is as short as that in *P*. *houi* and *Eopengornis martini*; the dorsal and ventral margins define an angle of approximate 20°. Both the maxillary process and the nasal process taper caudally. Four teeth are preserved in the left premaxilla, as in other enantiornithines; the right preserves three teeth, with the rostral one missing. Due to preservation, only two teeth are visible in the middle of the right maxilla. Compared to the conical and unrecurved teeth in *P*. *houi*, the teeth of *Parapengornis eurycaudatus* are smaller (crown height 0.5–0.55 mm), more slender and constricted towards the occlusal tip with slightly pointed and recurved apexes (Fig 2C and 2D). These teeth more strongly resemble those of *Eopengornis* than *Pengornis*, but differ from *Eopengornis* in being relatively less pointed and recurved (Fig 2E). The robust maxilla resembles that of *P*. *houi* with a well developed broad ascending process defining a 30° angle with the caudally tapered jugal process [7]; the premaxillary and jugal processes of the maxilla are subequal in the new specimen, whereas the premaxillary process is slightly less developed in *Eopengornis*. The jugal ramus is excavated dorsally by a groove that extends nearly to the rostral base of the nasal process; this groove is also present in *Pengornis*. The nasal process of the maxilla is rostrally lined by a medially recessed sheet of bone, which seems to be the elongate nasal; the maxilla appears to be perforated by a maxillary foramen that is larger and more rostroventrally located than that of *P*. *houi*. The dorsal margin of this process is also forked, as in *P*. *houi*; this fork is much more distinct in the new specimen, although potentially exaggerated by poor preservation. A strap-like bone displaced ventrally over the mandible may represent the jugal; close to this bone is a forked element identified as the quadratojugal. The caudodorsal ramus of the lacrimal appears reduced so that the lacrimal is L-shaped, rather than T-shaped as in *P*. *houi* [7] (Fig 2F and 2G); the craniodorsal ramus is very long, approaching the length of the ventral ramus. The dorsal margin of the lacrimal in *Parapengornis eurycaudatus* is weakly concave, lacking the strong concavity present in *P*. *houi* [7]. The ventral ramus is excavated as in *P*. *houi* and some other enantiornithines [6]. The unfused frontals are tapered rostrally and expanded caudally typical of ornithothoracines [18]. Breaks in the right frontal in some areas suggest that these bones were pneumatized. The parietals are unfused to each other, and much smaller and shorter than the frontals, typical of ornithothoracines. The quadrate is long and straight similar to that of *Longipteryx chaoyangensis* [19], whereas this element is bowed in other enantiornithines (e.g. *Rapaxavis pani* [20], *Longirostravis hani* [21], *Eoenantiornis buhleri* [22], *Pengornis houi* [7], and *Eopengornis martini* [6]). The mandibular processes are obscured, and the orbital ramus is broad and slightly tapered. The occipital region of the skull is poorly preserved, revealing no anatomical information.

The mandible preserves nearly the complete dentaries, and hyoid rami; the postdentary bones are poorly preserved. The exposed right dentary is straight, with a blunt rostral margin; the caudal margins of both dentaries are obscured and the shape of the articulation with the postdentary bones is unclear. Dentary teeth are numerous as in other pengornithids, with seven teeth preserved in the right, and nine preserved in the left. They are morphologically similar to the premaxillary and maxillary teeth, except slightly larger in size. The hyoid bones are slender and rod-like, with expanded articular facets at the rostral end.

Ventral to the mandibles are a pair of elongate, sheet-like bones that may represent the displaced palatines, or alternatively represent postdentary elements (Fig 2).

#### Vertebral Column and Ribs

The vertebral series is fairly complete. The neck consists of eight articulated cervical vertebrae preserved in articulation; the first vertebra is tentatively identified as the axis. The cranial four cervical vertebrae are short, as in *Eopengornis* and *Pengornis* IVPP V18632 with nearly equal length and width. The caudal four vertebrae become slightly more craniocaudally elongated, although still proportionately shorter, compared to the cervical vertebrae in the holotype of *Pengornis houi*, in which the cervicals are strongly elongated throughout the entire cervical series [7]. The prezygapophyses are as short as the postzygapophyses and project craniolaterally. The cranial articular surface of the sixth cervical is exposed and heterocoelous. Short and very low neural spines are present, approximately one third the length of the centrum and centered on the dorsal surface.

Eight thoracic vertebrae are preserved; the first three are in articulation with the cervical vertebrae but poorly preserved, while the caudal five vertebrae are nearly in articulation with the synsacrum. They bear high spinous processes and deep lateral excavations, with the length-to-width ratio of approximately 1.5, as in *Pengornis houi* [7].

The cranial portion of the synsacrum is overlapped by the ilia, with only the caudal three vertebrae visible; these are completely fused. The enlarged transverse processes gradually elongate distally and the distal tips become slightly expanded as in *Pengornis houi* [7]. In *P*. *houi* these expansions contact each another enclosing fenestra, but they remain separate in the new specimen; this potentially may be ontogenetic and subject to closure in this new specimen as well.

Seven to eight free caudals are preserved in articulation. The total length of the pygostyle is 8.4 mm, which is shorter than the width, and less than half the length of metatarsal III. A short and broad pygostyle characterizes the Pengornithidae, whereas other enantiornithines have proximally dorsally forked elongate distally tapered pygostyles with ventrolaterally projecting processes. The broad condition observed in *Parapengornis eurycaudatus* is extreme (with length to midpoint width ratio 1.8) even compared to other pengornithids (2.5 in *Pengornis houi* and 2.7 in *Pengornis* IVPP V18632), and most enantiornithines (over 3.5, e. g. 3.8 in *Rapaxavis*, 4.8 in *Boluochia* and 4.7 in *Sulacavis*). The pygostyle is also short in *Eopengornis*, but differs in shape with *Parapengornis eurycaudatus* and may be incomplete. A caudal vertebra appears partially incorporated into the pygostyle as the propygostylar vertebra; the transverse processes are still identifiable. Just caudal to this vertebra, the main body of the pygostyle expands in mediolateral width. The caudal margin is defined by two symmetrical convexities on either side of a midline concavity so that the pygostyle is roughly heart-shaped; this is also present in IVPP V18632. The dorsal surface of the body of the pygostyle is concave and bears two longitudinal ridges, centered on each half of the pygostyle. The deep incision on the caudal margin in *Parapengornis eurycaudatus* is not preserved in the rectangular-shaped short pygostyle of *Eopengornis* [6], and this margin is abraded in the holotype of *P*. *houi*. The characteristic enantiornithine craniodorsal fork is absent in *Parapengornis eurycaudatus*, as in other pengornithids.

The long dorsal ribs are curved, with several unfused uncinate processes. They are short, only reaching the adjacent rib; their caudal ends appear to bear small rounded expansions rather than being bluntly tapered as in other known birds. Four to five short and robust sternal ribs articulate with each side of the sternum. Several slender elements disarticulated near the pelvic region are identified as gastralia (Fig 1).

#### Sternum

The sternum is similar to that of *Pengornis* IVPP V18632 [10] and *Eopengornis* [6]; differences in the morphology of the rostral margin are due to poor preservation in the latter two specimens and the ontogenetic stage of the new specimen. The pengornithid sternum is unique among enantiornithines, although strongly resembling the condition in the basal enantiornithine *Protopteryx* [4]. In the new specimen the rostral margin is only weakly vaulted so that the craniolateral margin defines an obtuse angle of 98°. A small incision between two sternal plates extends approximately a quarter of the length of the sternal body. This incision is continuous, with a faint suture that extends another quarter of the sternal length before it is obscured by breakage. The entire body of the sternum is heavily pitted, indicating the periosteal surface was incompletely ossified. The costal margin is angled caudomedially, so that the lateral margin is weakly concave. The lateral trabeculae are directed caudally, strap-like with oval cross sections, and bear no large distal expansions as in other pengornithids and *Protopteryx* [4]. However, the distal fifth of the lateral process is slightly wider than the proximal portion, constituting a small but distinct expansion in *Parapengornis eurycaudatus*, similar to *Pengornis* IVPP V18632 [10]; this is absent in *Eopengornis* [6]. The periosteal surface of the lateral trabeculae appears more completely ossified compared to the body of the sternum. The left margin of the crushed xiphial region is nearly straight, indicating intermediate trabeculae and a xiphoid process were absent so that the caudal margin defines a wide V, as in other pengornithids and similar to *Protopteryx* [4].

#### Pectoral girdle

The completely preserved left scapula is 46.3 mm in length, with a laterally hooked acromion process, a feature unique to the Pengornithidae among the Enantiornithes [6]. The acromion process is slightly longer than the length of the glenoid facet. The surface of the scapular glenoid tapers caudally. The scapular shaft is short and straight; the distal end is tapered, whereas the distal margin is blunt in *Eopengornis* [6].

The well preserved right coracoid is strut-like and proportionately shorter than that of *Pengornis houi*, measuring approximately 50% of the length of the humerus compared to and 60% in the latter (length to distal width ratio 1.92 compared to 2.02 in *P*. *houi*). A procoracoid process is absent, as in other enantiornithines, and the acrocoracoid process is rounded and proximodistally aligned with the humeral and scapular articular surfaces as in other pengornithids and enantiornithines. The medial and lateral margins of the corpus are concave proximally and for most their length but convex distally, so that the straight sternal margin is expanded, as in *Eopengornis* [6].

The furcula is Y-shaped as in most enantiornithines, with an interclavicular angle approximately 70°, intermediate between *Pengornis houi* (65°) and *Pengornis* IVPP V18632 (75°). The clavicular rami are slender and lack the distinct slight medial convexity present in *P*. *houi*, rather being straight as in *Pengornis* IVPP V18632. The omal tips are sharply tapered at the distal end, different from the blunt omal tips present in *Eopengornis* [6]. The omal tips are also heavily pitted and striated indicating poorly ossified periosteal bone. The dorsal excavation that characterizes the enantiornithine furcula is limited to the proximal two-thirds of the rami. The distal tip of the hypocleidium is covered by the sternum; however, we estimate this process to be two-thirds the length of the rami, which is longer than that preserved in *P*. *houi* (less than 1/2), but similar to *Eopengornis*.

#### Forelimb

The forelimbs are nearly complete and articulated, with a ratio of 1.31 with the hindlimb (humerus + ulna + carpometacarpus / femur + tibiotarsus + metatarsal III); this is approximately equal to the intermembral index of *Pengornis houi* (1.35) [7].

The humerus is shorter than the ulna, as in other pengornithids. In caudal view, the humeral head is only slightly convex proximally, as compared to the condition in *Pengornis houi*, but more obviously convex than in *Pengornis* IVPP V18632. Although obscured by overlap, the capital incision and the ventral tubercle both appear weakly developed compared to *P*. *houi*; this may be due to the ontogenetic immaturity of IVPP V18687. The narrow deltopectoral crest resembles that of *Pengornis* IVPP V18632 and *Eopengornis*, and differs from *P*. *houi*. The crest is approximately half the width of the humeral shaft (approximately equal in *P*. *houi*) and extends for more than 1/3 the length of the humerus, ending abruptly distally (distal margin abraded in *P*. *houi*).

The ulna is robust and bowed, approximately 105% the length of the humerus, proportionately slightly shorter compared to other pengornithids (108% in *Pengornis houi*, and 107% in *Pengornis* IVPP V18632, and 112% in *Eopengornis*).The radius is straight, and the mid-shaft width ratio relative to the ulna is approximately 1.8. The ulnare is slightly larger than the radiale and is differentiated into dorsal and ventral rami of unequal length as in *Pengornis houi*.

The carpometacarpus is entirely unfused, and shorter than half the length of the ulna, as in *Pengornis* IVPP V18632 and *Eoenantiornis* [23]. The semilunate carpal is unfused to the major metacarpal, whereas there is nearly complete fusion between the semilunate carpal and the proximal ends of the major and minor metacarpals in *Pengornis houi* [7]; this lack of fusion is most likely due to the early ontogenetic stage of the specimen. The alular metacarpal is short and rectangular, measuring approximately 1/5 of the major metacarpal in length. The major metacarpal is much more robust than the slender and bowed minor metacarpal, and the latter extends farther distally than the former as in other enantiornithines. The intermetacarpal space is broader than that of *Pengornis* IVPP V18632 and *Eopengornis*, and extends from the distal end of the alular metacarpal to the distal end of the major metacarpal.

The alular digit of *Parapengornis eurycaudatus* distinctly reaches the distal end of the major metacarpal, compared to nearly reaching the distal end in *Pengornis* IVPP V18632 and *Eopengornis*. The first phalanx of the alular digit is slender; the ungual phalanx is approximately equal to that of the major digit in size, whereas the alular claw is larger and more recurved in *Pengornis* IVPP V18632 and *Eopengornis*; this digit is not preserved in *P*. *houi*. The first phalanx of the major digit is robust and rectangular with a slight constriction at the proximal third, and is longer than the penultimate phalanx. The first phalanx of the minor digit tapers distally towards its articulation with the extremely reduced second phalanx.

#### Pelvic girdle

The unfused pelvic elements are nearly complete and articulated, whereas only the flared distal ends of the pubes are preserved in *Pengornis houi* [7] and *Eopengornis* [6]. The unfused retroverted pubes are slightly shorter than the femur, with an expanded boot at the distal end of the pubic symphysis as in *P*. *houi* and *Eopengornis*. The preacetabular ala of the ilium is longer and dorsoventrally broader than the bluntly tapered postacetabular ala; the cranial end is ventrally expanded, also observed in some ornithuromorphs (e.g. *Archaeorhynchus* and *Piscivoravis*). The iliac pedicels are not laterally compressed, as in *Longipteryx*. The ischium is slender and a dorsal process is absent, which may characterize pengornithids.

#### Hindlimb

The hindlimb is nearly completely preserved. The femur is slightly craniocaudally bowed and approximately equal to the length of the tibia (a ratio of femur to tibia being approximately 99%), comparable to other pengornithids (*Pengornis houi* (95%), *Pengornis* IVPP V18632 (92%), and *Eopengornis martini* (90%)), and *Longipteryx* (94%) [19]. The femora in most other enantiornithines are distinctly shorter than the tibia with a ratio less than 90% (e.g. *Rapaxavis* [20] (81%), *Longirostravis* [21] (78%), *Sulcavis* [24] (87%), *Bohaiornis* [17] (85%), and *Vescornis* [25]. The femoral neck is not as obvious as in *P*. *houi*, and distally the medial condyle is larger than the lateral condyle.

In proximal caudal view of the tibia, the lateral articular facet and the popliteal tuberosity are developed, as in the holotype of *Eopengornis* and *P*. *houi*; however, in the new specimen they appear subequal, defining a more substantial flexor fossa. The fibula is long, nearly reaching the distal end of the tibia, as in other pengornithids. The distal end of the fibula is tapered and pointed, rather than bearing a small rounded expansion as in *Eopengornis* [6]. The astragalus and calcaneum are free from the tibia and each other, an artifact of ontogenetic immaturity.

**Fig 3:**
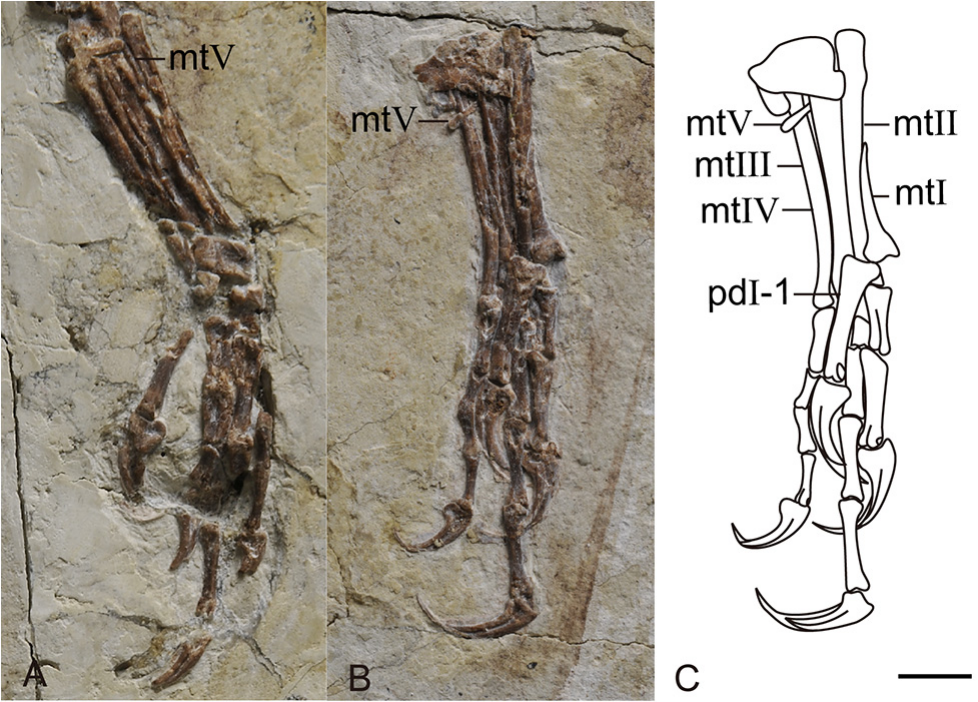
Detail photographs and line drawing of the feet of IVPP V18687 (A. right photograph; B. left photograph; C. left line drawing). Abbreviations: mtI-V, metatarsals I-V; pd I-1, pedal digit I-1. Scale bar equals 5 mm.

Metatarsals II-IV are entirely unfused to each other, similar to other enantiornithines. A thin element overlying the left metatarsal IV and another comparable element situated between the right metatarsals III and IV are identified as metatarsal V (Fig 3). Metatarsal V is a very short, thin, rod-like bone with a tapered distal end, measuring approximately 16% of the length of metatarsal II. Until now, this feature could only be definitely confirmed in *Eopengornis* [6] among enantiornithines, and unreported in any enantiornithine from the Jiufotang Formation. The P-shaped metatarsal I articulates with the medial surface of metatarsal II. Metatarsal I is elongate (42% the length of metatarsal III) comparable to *Eopengornis*, but longer than other pengornithids (33%), and other enantiornithines (25%) (Fig 3). The articular surfaces of metatarsal I are arranged in planes oriented at 90° so that despite the medial articulation with metatarsal II the hallux is fully reversed. The lateral trochlear ridge of metatarsal I is distinctly wider than the medial trochlear ridge, although the intertrochlear groove is very weak, possibly due to abrasion. The trochlea of metatarsal II and III are obscured by the hallux, but the position of the phalanges indicate that metatarsal III reached farther distally than metatarsals II and IV, which are subequal. Metatarsal IV is narrower than metatarsals II and III as in other enantiornithines [1].

The proximal phalanx of the hallux is robust and the longest in the foot, exceeding the length of metatarsal I and measuring approximately half the length of metatarsal III (Fig 3), as in *Eopengornis* [6]. In comparison, this phalanx is shorter than metatarsal I and less than 1/3 the length of metatarsal III in *Pengornis* IVPP V18632. The proximal phalanx of digit II is considerably shorter than the second phalanx, as in other pengornithids. In digit III the proximal phalanx is slightly longer than following two phalanges. The ungual phalanges in digit II and III are approximately equal in size, and larger than those of digits I and IV. The phalanges of digit IV are reduced and shorter and more slender than the other digits, typical of the foot in enantiornithines.

#### Plumage

This specimen preserves feather impressions throughout the skeleton, except in the hind limbs. The wings are long and pointed; the primaries (121.2 mm in the left and 144.2 mm in the right) are longer than the secondaries (91.2 mm in the left and 58.8 mm in the right). Preservation is not clear enough to determine the exact number of each type of remige feather that make up the wing. The tail consists of two distally incomplete elongate fully pennaceous rectrices; this morphology was previously only known in the older pengornithid *Eopengornis* [6], thus this specimen represents the first report of this morphology in the Jiufotang Formation. The rectrices measure 131.5 mm from the pygostyle to the distal preserved ends. As in *Eopengornis*, the faint impression of barbs can be identified along the entire length of the rectrices, whereas in *Confuciusornis* [26] and other enantiornithines (e.g. *Dapingfangornis* [27], *Paraprotopteryx* [28]) with elongate paired rectrices in the pennaceous portion is distally restricted as in the ‘racket-plumes’ of living birds. The medial and lateral barbs both form a small angle of approximately 10° with the rachis, and the lateral vane seems exceeds the width of the medial vane, although vaguely preserved in this impression, while far more obvious in *Eopengornis* STM 24–1 [6].

### Phylogenetic Analysis

**Fig 4:**
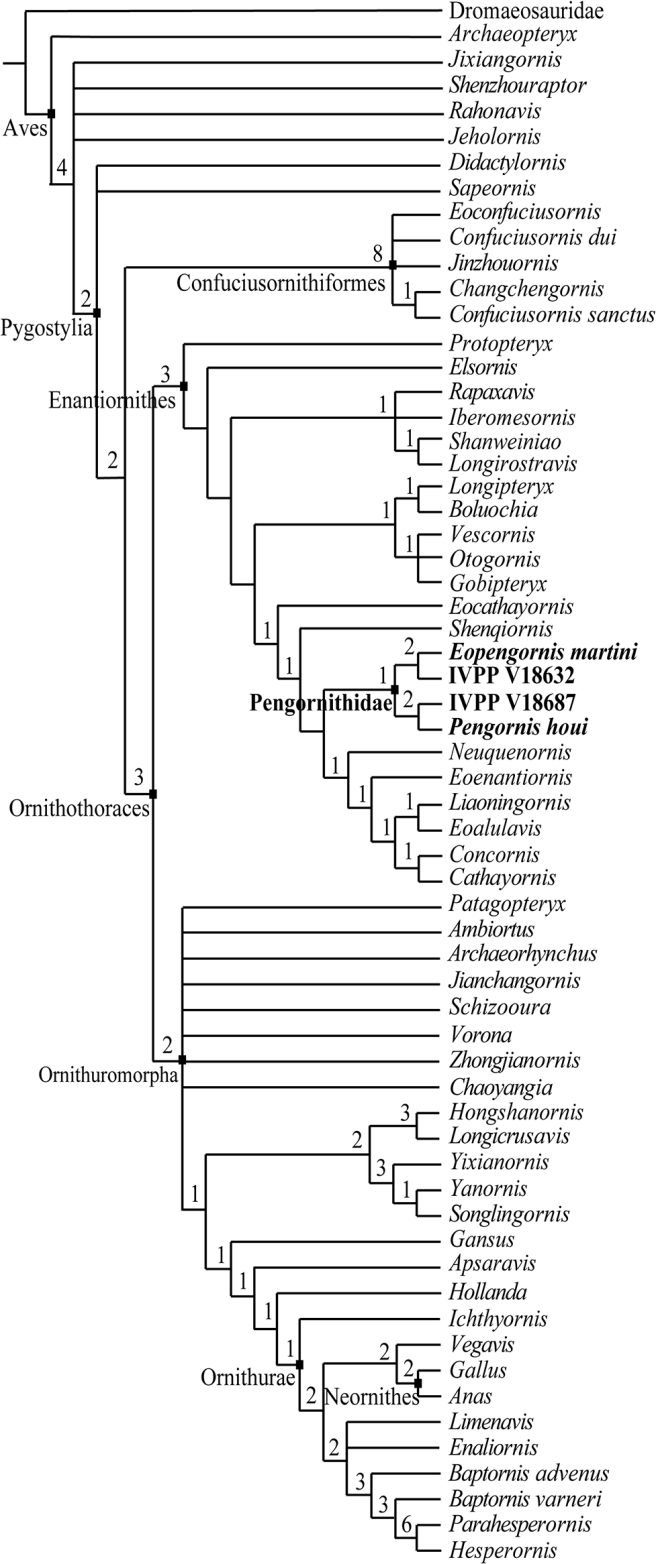
Strict consensus tree illustrating the phylogenetic position of *Parapengornis eurycaudatus*, and its close relationship with *Pengornis houi*, *Pengornis* IVPP V18632 and *Eopengornis martini* (tree length = 939 steps; CI = 0.366; RI = 0.673). Absolute Bremer support values are labeled at respective nodes.

The heuristic search in the analysis, implementing 1000 replications of tree branch-connection reconnection branch swapping (TBR) saving ten trees per replication, returned 100 most parsimonious trees (MPTs) 939 steps long. Another round of TBR branch swapping returned 564 MPTs. The strict consensus tree confirms morphological inferences that *Parapengornis* is a member of the Pengornithidae (Fig 4).

In the strict consensus tree, Enantiornithes is well resolved but weakly supported as indicated by the low absolute Bremer support values (most are 1) and the low Consistency Index (0.366) and Retention Index values (0.673). A monophyletic Longipterygidae [29] is not supported as in the result of O’Connor et al. (2012) [24], with its former members divided into two clades: *Rapaxavis*, *Shanweiniao*, and *Longirostravis* are resolved in a basal clade with *Iberomesornis*, while *Longipteryx* + *Boluochia* holds a more derived position.

The basal position of the Pengornithidae within the Enantiornithes resolved by several previous analyses is not supported in the strict consensus produced by this analysis [7–10]. *Parapengornis eurycaudatus* and other pengornithids (*Pengornis houi* [7], *Pengornis* IVPP V18632 [10], and *Eopengornis martini* [6]) are resolved as a clade, although absolute Bremer support for this node is low (value = 1). The analysis indicates several synapomorphies for this node, such as no distinct convex lateral coracoid margin (character 90: 0); no distal expansion of sternal outermost trabecula (character 113: 0); and caudal end of sternal midline V-shape (character 118: 1).

Ornithuromorpha is less resolved than the Enantiornithes. It resembles previous results [9] in the polytomy of the most basal taxa, and the relative positions of the Hongshanornithidae, Songlingornithidae and other derived taxa; the new results differ in that *Ambiortus* is resolved in a more basal position, rather than being resolved with *Apsaravis* in a more derived clade [9] (Fig 4).

### Histology

**Fig 5:**
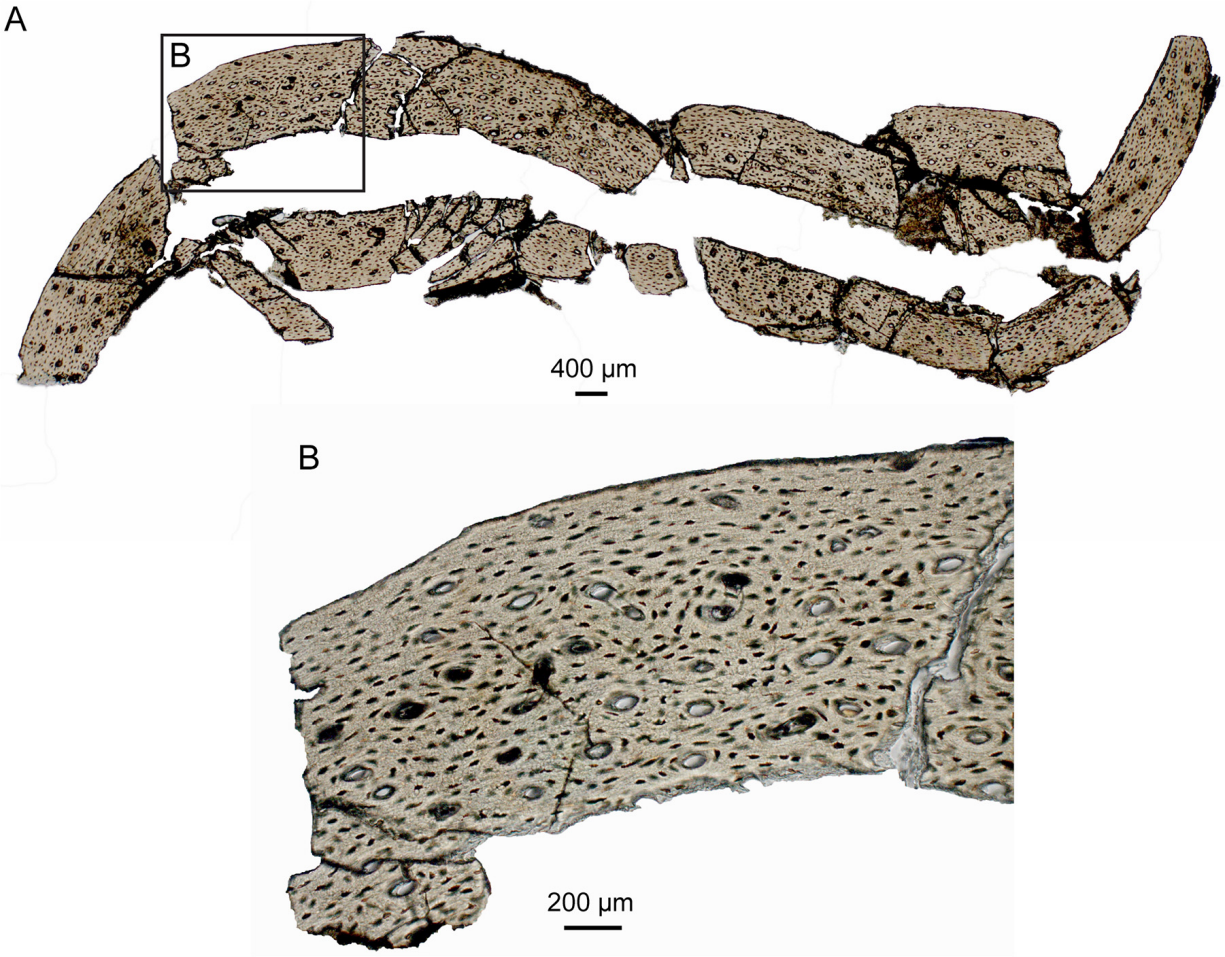
Histology of the femur of IVPP V18687: A, entire section; B, close up image of the section.

The midshaft portion of the femur of IVPP V18687 was sampled for histological analysis (Fig 5A). Following the methodology and terminology of Chinsamy [30], the sample was embedded in resin, then cut and polished after being allowed to dry for 24 hours.

The bone wall of the femur consists mainly of woven-texured bone tissue, with no distinctive stratification into the inner circumferential layer (ICL), middle region, and outer circumferential layer (OCL) (Fig 5B) that is typical of adult Jehol enantiornithines [31]. The absence of an ICL indicates this specimen is immature, representing a late stage juvenile or very young subadult. The compacta can be weakly distinguished into two regions: a thick inner region with plump, haphazardly organized osteocyte lacunae and abundant primary osteons, and an outer region with relatively flatter and more parallel arranged osteocyte lacunae. No secondary osteons or lines of arrested growth (LAG) are visible, indicating that IVPP V18687 probably had not lived a full year at the time of death. Although no distinctive OCL is developed, the osteocyte lacunae in the outer region near the exterior margin of the bone wall are all flat and have a parallel arrangement to the bone surface, suggesting growth had somewhat slowed.

The bone tissue of the holotype of *Eopengornis martini* STM 24–1 (a subadult individual) shows that the ulna was still growing at a relatively fast rate, while the rate of deposition of bone tissue in the femur and the humerus had relatively slowed [6]. Comparing the bone tissue from the ulna of *E*. *martini* STM 24–1, in which an ICL is present, IVPP V18687 can be interpreted as younger than STM 24–1, having yet to undergo later remodeling of the bone tissue. However, there is no trace of fibrolamellar bone like that observed in one embryonic enantiornithine [32], which may suggest that rapidly formed embryonic bone tissue had been resorbed.

### Ontogenetic Status

The new specimen is estimated to weigh 163.3g [33], smaller than the ‘adult’ holotype specimen of *Pengornis houi* [7], which is estimated to be 235.1g. Apart from its smaller size, the new specimen is clearly a young subadult, based on the incomplete fusion of the sternal midline, and the absence of fusion in the carpometacarpus, tibiotarsus and tarsometatarsus. In addition, the periosteal surface is incompletely ossified in many elements (in order of least ossified to most completely ossified): sternum, furcula, scapula, coracoid, humerus, ulna, pubis, femur and tibiotarsus. The surface of the sternal body is entirely porous, while most other elements bear sparse longitudinally arranged pits. The omal tips of the furcula and distal ends of the pubis bear dense pits and striations.

These features are consistent with the histology of the femur of IVPP V18687, which indicates it was ending its rapid growth phase at the death time. Major bone remodeling (e.g. medullary expansion and the deposition of an ICL) had yet to occur, and based on the absence of growth lines, the individual was less than one-year old. Enantiornithines apparently took several years to reach skeletal maturity [31, 34]; based on comparison with a previously sampled enantiornithine from the Jehol [31], we consider that IVPP V18687 had yet to reach sexual maturity.

## Discussion

### Pengornithid Taxonomy

*Parapengornis eurycaudatus* shares several diagnostic features with previously known pengornithids *Pengornis houi* [7], ‘*Pengornis*’ IVPP V18632 [10], and *Eopengornis martini* [6], that readily allow its referral to the Pengornithidae: premaxillae relatively short and entirely unfused; teeth extremely small and numerous; pygostyle short and broad; tip of the scapular acromion hooked; sternum with a V-shaped caudal margin and intermediate trabeculae absent; ulna 110–115% length of the humerus; femur approximately equal to tibia in length; elongated fibula nearly reaching the distal end of the tibia; and elongated metatarsal I and digit I-1. This is supported through phylogenetic analysis (Fig 4).

Apart from the strong resemblance between *Parapengornis eurycaudatus* and other pengornithids, there exist substantial morphological differences that indicate despite its ontogenetic immaturity IVPP V18687 cannot be referred to any previously known pengornithid species and represents a new member of this clade. In *Parapengornis eurycaudatus*, the teeth are numerous and small as in other pengornithids, but more slender with pointed and recurved apexes, rather than short and conical with blunt and unrecurved apexes as in *P*. *houi*, but relatively less recurved and tapered when compared to *E*. *martini*. The lacrimal is L-shaped with a strongly reduced caudal ramus, rather than T-shaped with nearly equal cranial and caudal dorsal rami as in *P*. *houi*. The cranial cervicals are short with only the caudal portion of the series slightly elongated, whereas the vertebrae are strongly elongated throughout the entire cervical series in *P*. *houi*. The pygostyle is proportionately shorter, being less than half the length of metatarsal III (41% of the latter in length), whereas it is longer in *P*. *houi* (63%) and other enantiornithines (e.g. 110% in *Rapaxavis* [20], 123% in *Boluochia* [35], 120% in *Longipteryx* [19], 79% in *Sulacavis* [24], 82% in *Bohaiornis* [17]), with the exception of *E*. *martini* (29%; may be incomplete). The lateral processes of the pygostyle are strongly expanded and the caudal margin forms two lobes, whereas this morphology seems much weaker in *P*. *houi* and *E*. *martini*. Metatarsal I is strongly elongated and nearly half the length of metatarsal III, whereas this ratio is approximately 1/3 in *P*. *houi*. Pedal phalanx I-1 is longer than metatarsal I and approximately half the length of metatarsal III. These characters are not considered subject to ontogenetic change at this stage in development, and therefore despite the fact this specimen is considered a young subadult, we erect a new taxon of the Pengornithidae for IVPP V18687, *Parapengornis eurycaudatus* gen et. sp. nov.

Comparison with the previously reported pengornithid IVPP V18632 suggests that it should be referred to *Parapengornis eurycaudatus*, although this is not supported by the current phylogenetic analysis. Previously this specimen was identified as a species of *Pengornis* on the basis of features that are now recognized to be synapomorphies of Pengornithidae, e.g. the entirely unfused premaxillae; numerous small teeth; the hooked scapular acromion; femur approximately equal to tibia in length; and the elongated fibula [10]. In light of comparison with the new specimen described here, we recognize the presence of several shared diagnostic features that suggest a closer affinity to *Parapengornis eurycaudatus*, while distinguishing it from *Pengornis*, including: furcula with slender and straight clavicular rami, rather than robust with distinct slight medial convexity in *P*. *houi*; cranial cervicals short but caudal cervicals more elongated, rather than strongly elongated throughout the entire cervical series as in *P*. *houi*; broad pygostyle shorter than half of metatarsal III with strongly expanded lateral processes and midline concave caudal margin, rather than elongated up to 2/3 of metatarsal III in *P*. *houi*, although the caudal margin is abraded in the holotype and may also be lobed in this taxon. Based on the shared features between V18632 and the holotype of *Parapengornis eurycaudatus*, and observable differences with the holotype of *P*. *houi*, we reassign IVPP V18632 to *Parapengornis eurycaudatus*.

Pengornithidae has previously been inferred to be a basal clade within Enantiornithes [6–10]. The first discovered pengornithid *Pengornis houi* [7] is the largest known Early Cretaceous enantiornithine, estimated to weigh 235.1g [33]; the smallest pengornithid, the holotype specimen of *Eopengornis martini* (STM 24–1), is estimated to weigh 94.5g but this is not considered the full adult body size. *Parapengornis* IVPP V18632 and IVPP V18687 are estimated to be 130.1g and 163.3g respectively, which although smaller than *P*. *houi*, is still larger than most other enantiornithine birds (according to the estimation of Liu et al., 2012 [33], most enantiornithines weigh less than 100g). As with STM 24–1, the adult mass of *Parapengornis* is expected to be greater than observed in these two specimens.

The oldest pengornithid *Eopengornis martini* weighs less than *Parapengornis* IVPP V18687 despite the relatively greater degree of ontogenetic maturity in the holotype specimen of the former; this suggests a trend towards increased body size within the Pengornithidae. Until the discovery of additional pengornithids, the basal position of *Pengornis houi* led to the hypothesis that basal enantiornithines were large, similar to more primitive birds (e.g., *Jeholornis*, *Sapeornis*, *Confuciusornis*), and that enantiornithines expanded their total size range to include both smaller and larger forms as they refined their apparatus [17, 29, 36]. The small size of the basal and stratigraphical lower *Eopengornis* invalidates this hypothesis. The basal position of the Pengornithidae is inferred morphologically by several primitive features, such as an elongate fibula (nearly reaching the distal end of the tibia), primitive sternum (intermediate trabeculae absent), and a metatarsal V (clearly preserved the holotypes of *Parapengornis eurycaudatus* and *Eopengornis*). However, the basal position of this clade is not supported by the present phylogenetic analysis. We hypothesize that this reflects inadequate character sampling for the Ornithothoraces, reflected by the weak support resolved for most nodes (Fig 4).

### Habitat of Pengornithidae

This new specimen preserves the most informative pengornithid pygostyle known thus far, the morphology of which hints at a specialized arboreal habitat for the Pengornithidae. The unique pygostyle of this clade is reminiscent of that present in extant woodpeckers, which may suggest pengornithids had the ability to climb and cling to vertical tree surfaces, an ecology that has not previously been inferred for Cretaceous birds.

Among neornithines, woodpeckers are extremely well-adapted for a specialized arboreal life style of climbing and clinging to vertical tree trunks. In extant woodpeckers morphological adaptations for this unique scansorial habitat are primarily reflected in three aspects: the zygodactyl arrangement of the pedes, the enlarged pygostyle, and the stiffened tail feathers [37]. *Parapengornis eurycaudatus* shares with woodpeckers an expanded pygostyle and potentially stiffened tail feathers. Although lacking the zygodactyl pedal arrangement, the pedal morphology of *Parapengornis eurycaudatus* and other pengornithids suggests more advanced arboreal capabilities relative to most other enantiornithines. Therefore, we explore the similarities between pengornithids and woodpeckers in these three regards, and discuss the possibility of such a specialized scansorial ecology characterizing the Pengornithidae.

Modern woodpecker-like birds are characterized by a zygodactyl pedal morphology: the second and third toes point forward, while both the first and fourth toes are reversed [37, 38]. This feature is absent in the Pengornithidae and no reliable direct evidence presents in any Mesozoic bird so far. *Parapengornis eurycaudatus* possesses the typical anisodactyl foot morphology (only the first digit is reversed), but evidence from modern woodpeckers indicates that different foot types may not be strictly correlated with ecology, and birds with different foot types may independently adapt to the same ecological niche [38], e.g. the anisodactyl foot type in the climbing and clinging Brown Creeper (*Certhia americana*).

Like other enantiornithines, *Parapengornis eurycaudatus* is inferred to be arboreal based on the presence of a reversed hallux and large recurved claws [1]. However, in pengornithids and most basal birds, the trochlea of metatarsal III extends beyond that of the other metatarsals, whereas in modern arboreal birds the metatarsals end at approximately the same level [39, 40]. In living arboreal birds the penultimate phalanx is also notably longer than the preceding phalanges in each digit [41]; in *Parapengornis eurycaudatus* this condition is present in digits II and IV, but absent in digit III and thus the phalangeal proportions of digit III in *Parapengornis eurycaudatus* (III-1: 34.2%; III-2: 32.6%; III-3: 33.2%) are not characteristic of living arboreal birds. This indicates that although *Parapengornis eurycaudatus* may have been arboreal, like other enantiornithines and basal birds (except for *Rapaxavis*, which had achieved increased arboreal capabilities as reflected by its pedal proportions [42]), arboreal capabilities may have been limited relative to neornithines.

**Fig 6:**
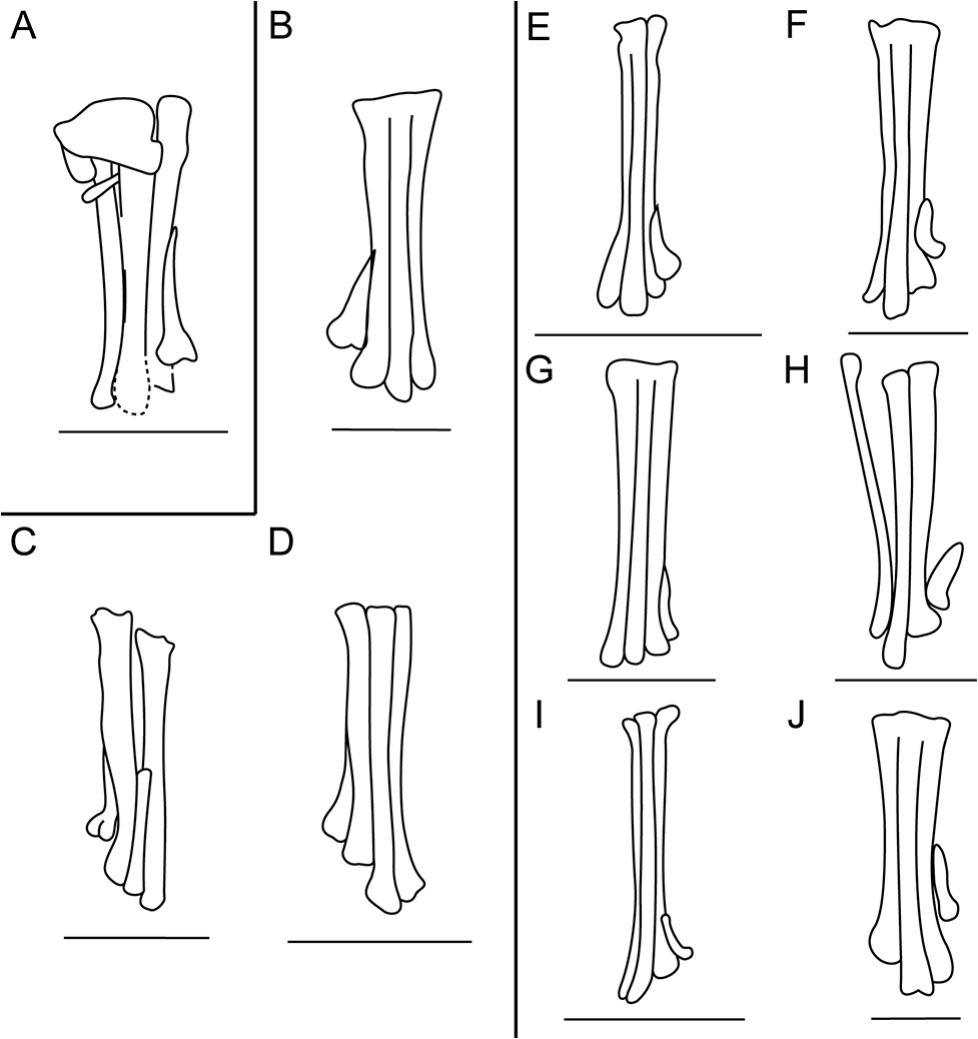
Comparison of metatarsals of A, IVPP V18687; B, *Pengornis houi*; C, *Pengornis* IVPP V18632; D, *Eopengornis martini*; E, *Rapaxavis pani*; F, *Sulcavis geeorum*; G, *Longipteryx chaoyangensis*; H, *Bohaiornis guoi*; I, *Vescornis hebeiensis*; J, *Confuciusornis yandica*. Scale bar equals 1 cm.

Despite these limitations, pengornithids display a combination of features that indicate increased arboreal capabilities relative to other enantiornithines. All members of the Pengornithidae possess a short tarsometatarsus relative to other enantiornithines (tarsometatarsus to femur ratio < 55%, compared to > 60% in most other enantiornithines, e.g. 70% in *Longirostravis* [21] and *Dapingfangornis* [27]), which increases stability while perching by lowering the center of mass [43]. A short and robust foot is also inferred to be an adaptation to a climbing and clinging life and is also observed in woodpecker-like scansores. Metatarsal I in *Parapengornis eurycaudatus* and other pengornithids is more than 30% the length of metatarsal III, which is proportionately longer than other enantiornithines (Table 2; Fig 6). Pengornithids also have an elongate and robust hallux (digit I phalanx 1 is longest in the pes), which further suggests enhanced grasping abilities. Notably, an elongate metatarsal I and hallux are also present in some modern woodpeckers, e.g. the highly specialized Ivory-billed woodpecker [38].

**Table 2:** Comparison of length ratio of metatarsal I to metatarsal III of pengornithids and other enantiornithines.

**Pengornithids**	***Parapengornis***		***Pengornis***		***Eopengornis***	
**Metatarsal I / III length ratio (%)**	42		33		47	
**Other enantiornithines**	***Rapaxavis***	***Longipteryx***	***Bohaiornis***	***Boluochia***	***Vescornis***	***Sulcavis***
**Metatarsal I / III length ratio (%)**	25	25	25	23	23	20

**Fig 7:**
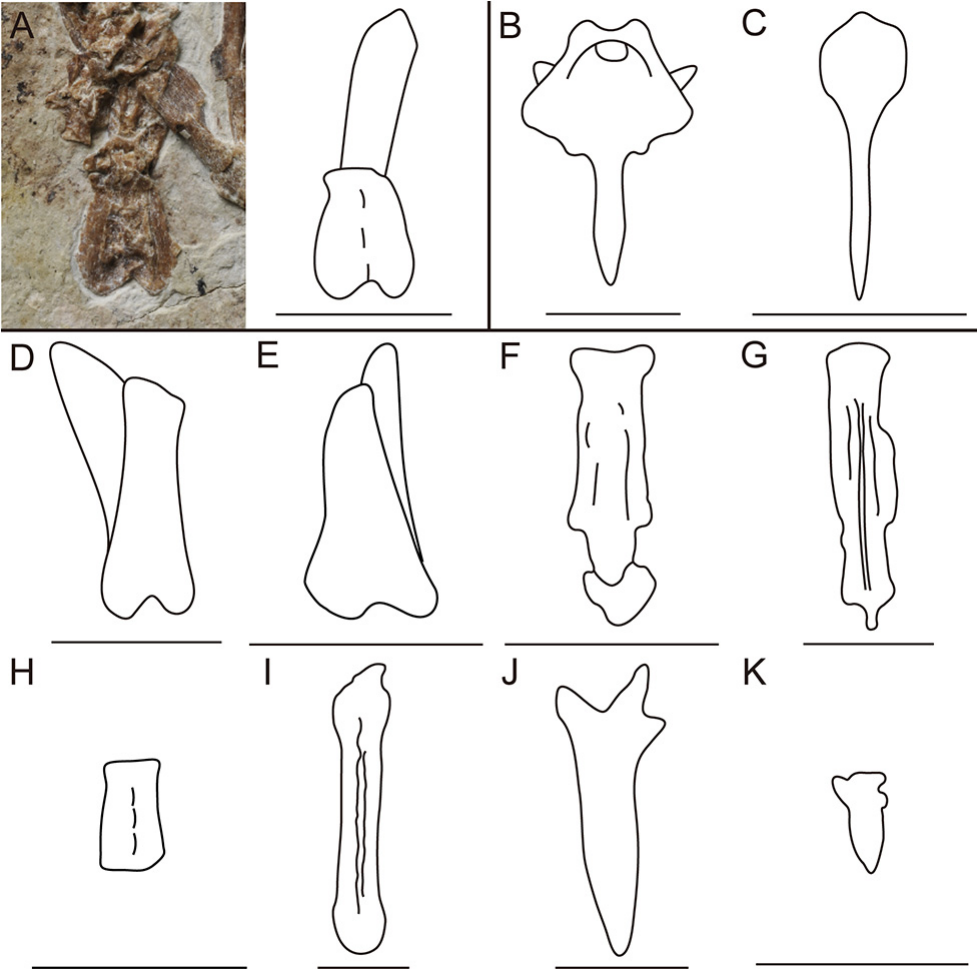
Comparison of pygostyles of A, IVPP V18687; B, *Picus sp*.; C, *Corvus splendens*; D, *Pengornis houi*; E, *Pengornis* IVPP V18632; F, *Rapaxavis pani*; G, *Boluochia zhengi*; H, *Eopengornis martini*; I, *Confuciusornis yandica*; J, *Sulcavis geeorum*; and K, *Archaeorhynchus spathula*. Scale bar equals 1cm.

In addition to their derived foot, woodpeckers use their tail feathers as a prop to provide additional support during vertical climbing and clinging on tree trunks. The unique function of the tail feathers is reflected in the extremely specialized morphology of the pygostyle and the appearance of novel features such as the lamina and discus pygostyle [37, 44]. The woodpecker pygostyle has a distinct laminar-shape, with the ventral surface bearing large lateral expansions (lamina pygostyli), which serve to increase the surface area available for attachment of the rectrices and enlarged tail depressor muscles (Fig 7B) [37, 38, 45]. The cranial end of the ventral surface is deeply concave (discus pygostyli), and the lateral expansions form a diamond-shaped outline, tapering to a point caudally (Fig 7B). In dorsal view, the dorsal crest is dorsoventrally expanded, and the transverse processes are wide and cranially inclined [37].

A recent study on the caudal morphology in diving birds indicates that the elongate and straight shape of their pygostyle is an excellent predictor of diving locomotor behavior [44]. In accordance, the Cretaceous diving bird *Baptornis* possesses an elongate pygostyle [46], supporting the ecological predictive function of pygostyle shape in the fossil record. The lateral expansion of the lamina pygostyle is strongest in species of woodpeckers that more frequently use the tail as a prop, thus this morphology is correlated with tail feather function in woodpeckers [37, 38, 45]; this suggests that expanded pygostyle morphology may be used to help reconstruct the ecology of extinct birds with similar morphologies.

Early bird pygostyles fall into three major morphological types: short, plough-shaped and tapered, as in *Sapeornis* and Ornithuromorpha (e.g. *Archaeorhychus* (Fig 7K)), similar to most neornithines (e.g. *Corvus* (Fig 7C)); robust, proximally forked and distally constricted in most of the Enantiornithes (e.g. *Rapaxavis* (Fig 7F), *Boluochia* (Fig 7G), *Sulcavis* (Fig 7J)); and robust and rod-like in *Confuciusornis* (Fig 7I). The pygostyle of *Parapengornis eurycaudatus* is distinct among Cretaceous birds for its brevity and strongly expanded lateral processes (Fig 7A), which resembles the morphology of modern woodpeckers. The laterally expanded pygostyle morphology in woodpeckers similarly represents a distinct outlier among extant birds [37] (Fig 7B). The Brown Creeper (*Certhia americana*), a non-piciform bird with a climbing and clinging lifestyle similar to that of woodpeckers, also exhibits an expanded pygostyle, further supporting a relationship between this pygostyle morphology and a specialized vertical climbing habit. Thus, the laterally enlarged pygostyle in *Parapengornis eurycaudatus* may indicate pengornithids had a specialized scansorial habitat similar to woodpeckers and the Brown Creeper. At the very least, this pygostyle morphology suggests the presence of increased caudal musculature in the Pengornithidae.

In addition to their autapomorphic pygostyle morphology, the rectrices in the Picidae are specialized with reinforced rachises and stiffened vanes present in all but the outer two rectrices [37, 44]. The distal ends of the stiffened feathers are pressed against the vertical surface, serving as a prop to aid the feet in supporting the body against the downward force of gravity [47, 48]. In the most basal taxa only the medial pairs of rectrices are stiffened and the lamina pygostyle is expanded, but the discus pygostyle is lacking. By contrast, in derived taxa only the lateral pair of rectrices is unmodified and both the lamina and the discus pygostyli are well developed, indicating that tail feathers coevolved with the expanded pygostyle [37, 44]. In woodpeckers the tail feathers have a fan-shaped arrangement; this is absent in *Parapengornis eurycaudatus*, which instead preserves a single pair of fully pennaceous rachis-dominated rectrices like those present in the basal *Eopengornis* [6]. Because the feathers are rachis-dominated, it is possible they were capable of providing support similar to the stiffened feathers in woodpeckers. The rachis dominated morphology was originally hypothesized to have aided in a scansorial lifestyle [49]. Because of the inferred cost in terms of keratin and energetic investments, the presence of an enlarged rachis initially hinted at some specialized functional significance (balance, defense) as a secondary derived morphology [49]. Until now, no credible function has been proposed. The fully pennaceous morphology observed in *Parapengornis* and *Eopengornis* is inferred to represent a basal condition in the evolution of the ornamental ‘racket-plumes’ present in many enantiornithines and *Confuciusornis* [6]. Until the discovery of *Eopengornis*, all complete rachis-dominated feathers had a racket-plume morphology (only pennaceous distally), the length and morphology of which did not support early hypotheses that these feathers may have aided in balance or had any functional significance. However, the presence of rachis-dominated feathers in conjunction with the specialized woodpecker-like pygostyle morphology of *Parapengornis eurycaudatus* may suggest that the enlarged rachis fortified the feathers so they could be used for a support function, similar to the stiffened tail feathers in woodpeckers. We suggest that any support function would have been lost in lineages that evolved distally restricted barbs under sexually driven selection, consistent with the absence of associated features of the pygostyle and the increased relative length of these feathers. If rachis-dominated feathers originally evolved with a functional significance, this could offer an explanation for their presence in juvenile enantiornithines prior to both skeletal and sexual maturity (appearing after sexual maturity is reached in living birds): these feathers may plesiomorphically appear early in ontogeny consistent with their original functional role in precocial enantiornithines.

A specialized scansorial habitat for the Pengornithidae is supported by the unique pygostyle morphology similar to modern woodpeckers; this in turn provides evidence for a potential support function for the rachis-dominated rectrices in the holotype of *Parapengornis eurycaudatus*. This discovery suggests greater ecological diversity for Early Cretaceous enantiornithines than previously hypothesized, that they may have occupied very specialized ecological niches with diversified locomotive differentiation.

## Supporting Information

S1 Character List Character list used in the phylogenetic analysis in this study. (PDF)

S1 Dataset Matrix used in the phylogenetic analysis in this study. (TNT)
